# Association between type of bystander cardiopulmonary resuscitation and survival in out-of-hospital cardiac arrest: A machine learning study

**DOI:** 10.1016/j.resplu.2022.100245

**Published:** 2022-06-14

**Authors:** Matilda Jerkeman, Peter Lundgren, Elmir Omerovic, Anneli Strömsöe, Gabriel Riva, Jacob Hollenberg, Per Nivedahl, Johan Herlitz, Araz Rawshani

**Affiliations:** a Department of Clinical and Molecular Medicine, Institute of Medicine, Sahlgrenska Academy, University of Gothenburg, Gothenburg, Sweden; b Prehospen – Centre for Prehospital Research, University of Borås, Sweden; c Region Västra Götaland, Sahlgrenska University Hospital, Department of Cardiology, Gothenburg, Sweden; d Department of Clinical Science and Education, Center for Resuscitation Science, Solna, Karolinska Institutet, Sweden; e The Swedish Cardiopulmonary Resuscitation Registry, Centre of Registries, Västra Götalandsregionen, Gothenburg, Sweden

**Keywords:** Cardiac arrest, OHCA, Compression only CPR

## Abstract

**Aim:**

In the event of an out of hospital cardiac arrest (OHCA) it is recommended for a sole untrained bystander to perform compression only CPR (CO-CPR). However, it remains unknown if CO-CPR is inferior to standard CPR (S-CPR), including both compressions and ventilation, in terms of survival. One could speculate that due to the current pandemic, bystanders may be more hesitant performing mouth-to-mouth ventilation. The aim of this study is to assess the association between type of bystander CPR and survival in OHCA.

**Methods:**

This study included all patients with a bystander treated OHCA between year 2015–2019 in ages 18–100 using The Swedish Registry for Cardiopulmonary Resuscitation (SRCR). We compared CO-CPR to S-CPR in terms of 30-day survival using a propensity score approach based on machine learning adjusting for a large number of covariates.

**Results:**

A total of 13,481 patients were included (5,293 with S-CPR and 8,188 with CO-CPR). The matched subgroup consisted of 2994 cases in each group.

Gradient boosting were the best models with regards to predictive accuracy (for type of bystander CPR) and covariate balance. The difference between S-CPR and CO-CPR in all 30 models computed on covariate adjustment and 1-to-1 matching were non-significant. In the 30 weighted models, three comparisons (S-CPR vs. CO-CPR) were significant in terms of improved survival; odds ratio for men was 1.21 (99% confidence interval (CI) 1.02–1.43; Average treatment effect (ATE)); for patients ≥73 years 1.57 (99% CI 1.17–2.12) for Average treatment effect on treated (ATT) and 1.63 (99% CI 1.18–2.25) for ATE. Remaining 27 models showed no differences. No significances remain after adjustment for multiple testing.

**Conclusion:**

We found no significant differences between S-CPR and CO-CPR in terms of survival, supporting current recommendations for untrained bystanders regarding CO-CPR.

## Introduction

Each year approximately 6000 individuals suffer an out-of-hospital cardiac arrest (OHCA) in Sweden.^1^ The majority of OHCAs are due to cardiovascular diseases but other possible causes are trauma, intoxication, drowning, asphyxia, electrical accidents and suicide.^1^ Although there are more persons trained in CPR today than ever before, namely 5 million Swedish citizens i.e 50% of the total population, and despite the fact that the proportion receiving CPR before ambulance arrival has increased dramatically, from 31% in 1990 to 76% in 2018, survival rate in OHCA has remained unchanged around 10% during the last decade.^2^

Although the prognosis in OHCA is poor, it can be markedly improved if the patient receives adequate treatment in time as shown in large population-based studies in Denmark, Sweden and Japan.3., 4., 5. This includes cardiopulmonary resuscitation (CPR) and defibrillation. The beneficial effect of chest compressions on survival is well defined, however the importance of ventilations has been ambiguous.^6^ Before the year of 2010, guidelines for CPR outside hospitals performed by bystanders included both compressions and ventilation (standard CPR [S-CPR]). The current recommendation from the Swedish Resuscitation Council is to perform both compressions and ventilation, but in case of sole untrained rescuers, insecurity or when ventilation cannot be performed, or in case of dispatcher assisted CPR (DA-CPR) the recommendation is to perform only compressions (compression only CPR [CO-CPR]).2., 3.

We analyzed OHCA in Sweden to evaluate the association between type of bystander CPR and survival, using the Swedish Registry for Cardiopulmonary Resuscitation (SRCR). A previous study from the SRCR, reported that patients receiving compressions in combination with ventilation (i.e., S-CPR) were more likely to survive, compared to those only given compressions (i.e., CO-CPR).^7^ In the same study, both S-CPR and CO-CPR doubled the chances of survival compared to no bystander CPR. The authors suggested that the main reason for endorsing CO-CPR as an option was its association with higher overall CPR rates and thereby improved overall survival in OHCA. It is possible that due to the current pandemic, bystanders may be more hesitant performing mouth-to-mouth ventilation although this remains to be evaluated. The current investigation aimed to use contemporary data and a propensity score approach based on machine learning, to perform a head-to-head comparison of S-CPR and CO-CPR adjusted for all potential confounders and covariates.

## Methods

### Swedish registry for cardiopulmonary resuscitation

This was an observational cohort study using data available in the national quality registry; The Swedish Registry for Cardiopulmonary Resuscitation (SRCR). The registry has been collecting data on OHCA since 1990 in all 21 regions in Sweden and the coverage is at present almost 100%.^8^ Data recording is performed initially by the emergency medical services (EMS) online and later reviewed by a local coordinator who is trained by the SRCR. Data includes patient characteristics, most probable cause of the arrest including non-cardiac causes, location, type of CPR performed and witness status, among other variables (Table 1). Inclusion criteria in the SRCR are in line with the Utstein guidelines.^9^ A more detailed description of the registry has previously been published.^8^

**Table 1:** Characteristics of 13,481 patients with cardiac arrest in relation to type of bystander CPR.

	1CO-CPR	1S-CPR	2SMD
n	8188	5293	
Age – mean (SD)	69.48 (16.26)	67.39 (16.75)	0.126
Women – n (%)	2798 (34.2)	1711 (32.3)	0.039
Location of CA – n (%)			0.222
Home	6056 (74.1)	3394 (64.2)	
Public place	1438 (17.6)	1196 (22.6)	
Other place	681 (8.3)	698 (13.2)	
Most probable reason of CA – n (%)			0.133
Heart disease	5065 (63.4)	3358 (65.5)	
Overdose	297 (3.7)	165 (3.2)	
Accident/trauma	136 (1.7)	116 (2.3)	
Pulmonary disease	408 (5.1)	222 (4.3)	
Suffocation	251 (3.1)	138 (2.7)	
Suicide	152 (1.9)	144 (2.8)	
Drowning	31 (0.4)	53 (1.0)	
Other	1652 (20.7)	932 (18.2)	
Sports related CA – n (%)			0.133
No	5647 (96.4)	3647 (93.8)	
Regular exercise	152 (2.6)	198 (5.1)	
Elite sports	1 (0.0)	3 (0.1)	
Unknown	59 (1.0)	41 (1.1)	
CRITICAL TIME INTERVALS – median (IQR)			
Time from CA to EMS arrival	13.00 [9.00, 20.00]	15.00 [10.00, 22.00]	0.049
Time from CA to CPR start	2.00 [0.00, 6.00]	1.00 [0.00, 4.00]	0.105
Time from CA to first defibrillation	15.00 [10.00, 24.00]	15.00 [10.00, 25.00]	0.036
Time from CA to alarm	2.00 [1.00, 5.00]	2.00 [1.00, 5.00]	0.060
Witnessed status			0.094
Not witnessed	3131 (38.4)	1847 (35.1)	
Bystander	4981 (61.0)	3358 (63.8)	
Ambulance	19 (0.2)	13 (0.2)	
Other or combinations	29 (0.4)	47 (0.9)	
Bystander education level – n (%)			0.571
Laymen without CPR education	3391 (56.9)	1212 (30.8)	
Laymen with CPR education	1997 (33.5)	1825 (46.4)	
Professional	576 (9.7)	892 (22.7)	
Bystander profession – n (%)			0.332
No bystander CPR given	2 (0.0)	7 (0.2)	
Laymen	5870 (91.1)	3474 (79.5)	
Professional	575 (8.9)	891 (20.4)	
3DA-CPR – n (%)	5220 (66.0)	2599 (50.9)	0.310
Defibrillator connected by bystander – n (%)	506 (6.3)	801 (15.4)	0.298
Defibrillated by bystander – n (%)	159 (19.6)	342 (35.6)	0.364
STATUS AT EMS ARRIVAL – n (%)			
Consciousness	124 (1.6)	147 (2.9)	0.089
Palpable pulse	348 (4.5)	362 (7.1)	0.113
Breathing status			0.114
Normal breathing	216 (2.7)	251 (4.8)	
Agonal breathing	780 (9.6)	532 (10.1)	
No breathing	7100 (87.7)	4470 (85.1)	
Shockable rhythm – n (%)	1789 (22.2)	1332 (25.6)	0.082
Defibrillated – n (%)	2718 (33.4)	1967 (37.3)	0.081
Adrenaline need – n (%)	6744 (82.7)	4311 (81.6)	0.027
Amiodarone need – n (%)	1133 (14.0)	795 (15.1)	0.032
Intubated – n (%)	1811 (22.2)	1187 (22.5)	0.006
Hospitalized – n (%)	1703 (22.5)	1305 (26.6)	0.094
Treatment completed on scene – n (%)	262 (6.0)	261 (8.6)	0.099
IN-HOSPITAL MEASURES – n (%)			
3PCI			0.095
No	1148 (69.2)	825 (65.0)	
Yes	504 (30.4)	436 (34.3)	
Planned	7 (0.4)	9 (0.7)	
3CABG			0.069
No	1613 (97.6)	1224 (96.5)	
Yes	27 (1.6)	33 (2.6)	
Planned	12 (0.7)	11 (0.9)	
3ICD			0.041
No	1390 (84.3)	1043 (83.0)	
Yes	231 (14.0)	194 (15.4)	
Planned	28 (1.7)	20 (1.6)	
3CPC			0.143
No sequele	463 (71.6)	445 (74.9)	
Mild sequele	113 (17.5)	94 (15.8)	
Severe sequele	42 (6.5)	42 (7.1)	
Vegetative state	25 (3.9)	12 (2.0)	
Brain dead	4 (0.6)	1 (0.2)	

1 CPR = Cardiopulmonary Resuscitation; S-CPR = standard CPR; CO-CPR = Compression-only CPR.

2 SMD = Standardized mean difference. SMDs below 10% (0.1) are considered inconsequential.

3 DA-CPR = Dispatcher assisted CPR, PC I = Percutan Coronar Intervention, CABG = Coronary Artery Bypass grafting, ICD = Implantable Cardioverter Defibrillator, CPC-score = Cerebral Performance Category.

### Study population

**Fig. 1:**
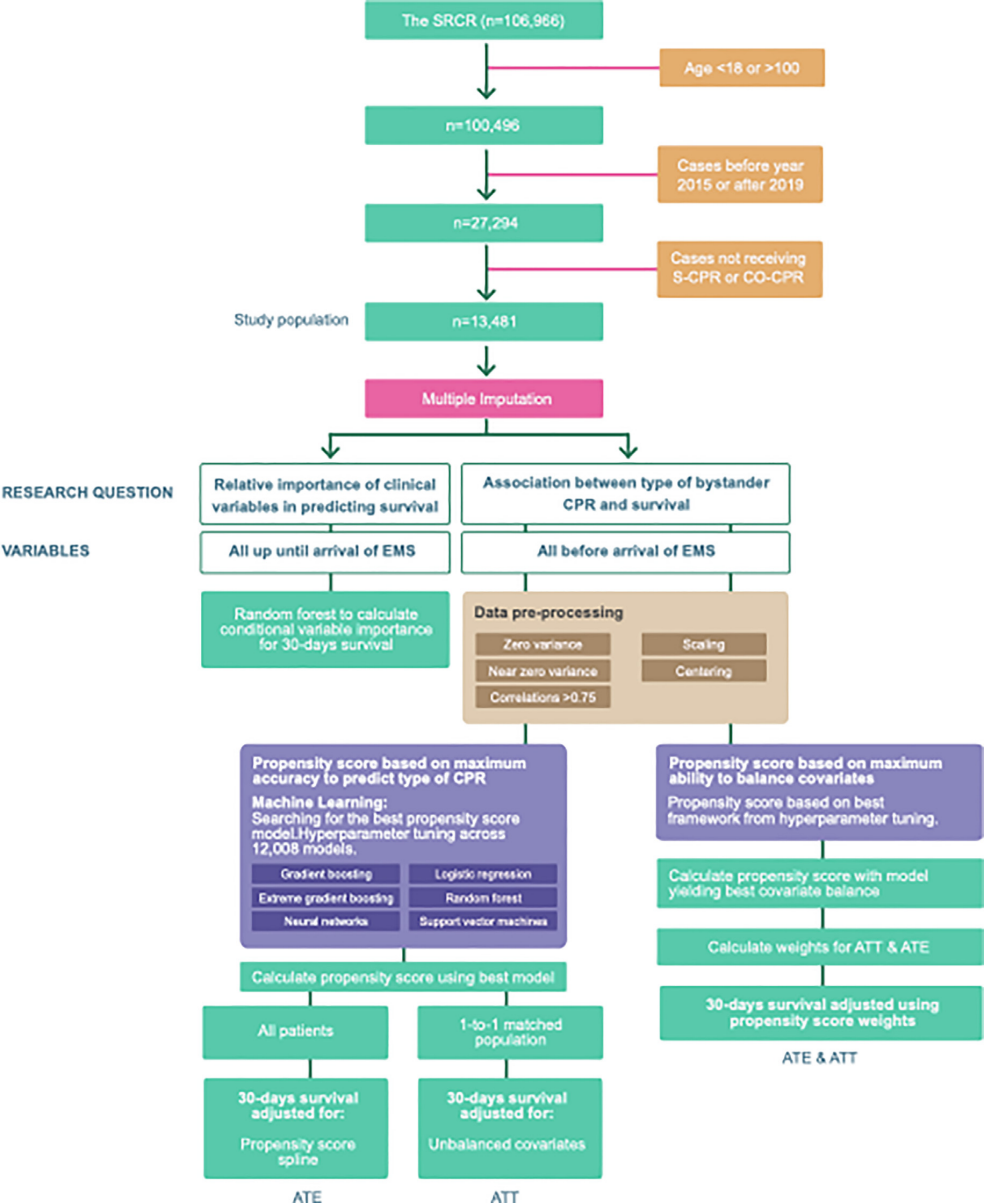
Flow chart over included and excluded patients, and an overview of the methods used. ATT (Average Treatment Effect), ATE (Average Treatment effect on Treated), EMS (Emergency Medical Services), compression only CPR (CO-CPR). Standard CPR (S-CPR), The Swedish Registry for Cardiopulmonary Resuscitation SRCR.

Included in the study were individuals 18–100 years of age, with bystander treated OHCA, provided that data on type of bystander CPR was available, during the time period 1 Jan 2015 to 31 Dec 2019. The study population was divided into the following two groups: standard bystander CPR with both ventilation and compressions (S-CPR) and bystander CPR with only compressions (CO-CPR). Refer to Fig. 1 for details.

Vital status is obtained on a daily basis by merging the SRCR with the Swedish population registry which has a complete level of ascertainment 30 days after death has occurred.

### Statistical analyses

The baseline characteristics were described using appropriate measures of central tendency (means, medians) and dispersion (standard deviation). To compare characteristics in the two groups standardized mean differences (SMD) was used.^10^ A statistically significant result was defined as an 99% confidence interval not crossing 1. Bar charts were used for visualizing distributions in location and cause of cardiac arrest.

The Kaplan Meier estimator was used to describe survival distributions; and log-rank test to compare the survival distributions. Kaplan-Meier curves were calculated in the overall group, as well as in men and women separately.

### Multiple imputation of missing data

In order to maximize power (i.e., the number of individuals with data on all predictors used in the prediction models) we used multiple imputation. We assumed that no mechanism explained missingness (i.e., data was missing at random).^11^ Missing values were imputed using the Multivariate Imputation by Chain Equations (MICE) algorithm.^12^ We used 50 iterations to impute one complete dataset, on which all prediction models were built and evaluated. Imputing multiple datasets was deemed as infeasible due to the large number of computationally intensive models that were planned, in addition to the fact that no confidence intervals were calculated using the imputed data. We used the built-in Predictive Mean Matching (PMM) method to impute missing values. PMM allows for flexible imputation of numerical and categorical data. In brief, PMM imputes missing values by evaluating a large number of prediction models and then use these models to predict the value of missing entries.

### Association between type of bystander CPR and 30-day survival

The association between type of bystander CPR and 30-day survival was studied using two different approaches (Fig. 1). In the first approach we used calculation of propensity scores using the model with highest accuracy for type of bystander CPR. In the second approach we used propensity score to calculate the inverse probability of treatment weighting (IPTW). The IPTW approach aims to balance the distribution of baseline covariates in the CO-CPR and S-CPR group by using weights based on the propensity score. Each observations weight is equal to the inverse probability of receiving the treatment actually received^13^ For further details regarding these approaches, we refer to Supplemental material.

### Relative variable importance for 30-day survival

In order to examine the importance of type of bystander CPR we calculated the relative importance of 23 early predictors of survival including type of bystander CPR, age, sex, calendar year, time from cardiac arrest (CA) to alarm, time from CA to CPR start, time from CA to EMS arrival, time from alarm to alert, time from alert to EMS arrival, sports related CA, reason for CA, location, location in a public place, location in other place, county, district, type of witness, bystander’s profession, bystander’s educational level, DA-CPR, defibrillator connected by bystander, clock time and weekday. Variable importance was calculated using a modified implementation of random forest, developed by Strobl et al.^14^ The original implementation of random forest is a highly efficient ensemble method that handles large numbers of observations and predictors and allows for complex modeling of interactions and non-linear functions. However, the original implementation is prone to yield biased estimates of relative importance for predictors that are correlated. Strobl and colleagues developed a conditional variable importance that allows for estimation of unbiased variable importance; such that the obtained estimates are not biased by correlations among the predictors. This analysis only included variables that can be assessed up until EMS arrival. The purpose of restricting the variable inclusion in this analysis is to provide information on which variables are the strongest predictors upon EMS arrival to the scene.

All analyses were done in R 4.0.3 (R Foundation for Statistical Computing [https://www.r-project.org]). The study has been approved by the Swedish Ethical Review Authority (approval ID 2019-01094).

## Results

A total of 106,966 patients were recorded in the SRCR during 1990–2019. After excluding patients under 18 and over 100 years of age, excluding patients enrolled before 2015 or after 2019, and requiring that they be treated with S-CPR or CO-CPR before arrival of EMS, our study population consisted of 13,481 patients (Fig. 1).

### Patient characteristics

The groups S-CPR and CO-CPR consisted of 5,293 and 8,188 patients, respectively (Table 1). In the S-CPR group the mean age was 67.4 years, the percentage of women was 32.3% and the most common location for CA was at home (64.2%). Heart disease was the most common etiology (65.5%) for the cardiac arrest. In the CO-CPR group, the mean age was 69.5 years, the percentage of women was 34.2% and the most common location for the CA was at home (74.1%). The most common etiology was heart disease (63.4%). The percentage of patients with a CA during exercise was slightly higher in the S-CPR group. There were no considerable differences in time from CA to EMS arrival, time from CA to first defibrillation or time from CA to alarm between the groups. Time from CA to CPR start was somewhat longer in the CO-CPR group. DA-CPR was more frequent in the CO-CPR group (66.0% vs 51.9%). In the CO-CPR and S-CPR groups defibrillation by bystanders were performed in 19.6% and 35.6% respectively. Further details about patient characteristics are described in Table 1.

### Survival distribution

The unadjusted 30 days survival when including all patients was higher in the S-CPR group (15%) compared to the CO-CPR group (11%), *P*-value <0.0001. Among men the 30 days survival rate was higher in the S-CPR group (18%) compared to the CO-CPR group (13%), *P*-value <0.0001. In women the 30 days survival was similar in the two groups (8%), *P*-value 0.058. Refer to Supplementary Fig. 1 for details.

### Relative importance of early predictors of survival

This model only included predictors that can be assessed immediately on EMS arrival. The analysis showed that the strongest predictor of 30 days survival, was breathing status, followed by initial rhythm, age and pulse at EMS arrival. Witnessed status, location, etiology of the CA, time from CA to defibrillation, time from CA to EMS arrival, consciousness at EMS arrival and bystander connected defibrillator were also shown to be important for predicting survival (Supplementary Fig. 2).

### Propensity score models and odds ratios for 30-day survival in relation to type of bystander CPR

#### Propensity scores for covariate adjustment and 1-to-1 matching

In total we built 12,008 different models using Gradient Boosting Machine (GBM), Support Vector Machine (SVM), Random Forest (RF), Logistic Regression (GLM), Extreme Gradient Boosting (XGBOOST) and neural networks. We compared the models using accuracy. We found the highest mean accuracy (0.665) for the GBM model (Supplementary Fig. 3). Supplementary Table 1 shows baseline characteristics of cases included in the 1-to-1 matched subgroup. The matching allowed for satisfactorily balancing of all variables with the exception of educational level of bystanders, DA-CPR and whether the bystander connected a defibrillator; these covariates were therefore included in the logistic regression model. A graphical summary of all GBM models’ accuracies across the hyperparameter grid is presented in Supplementary Fig. 4.

**Fig. 2:**
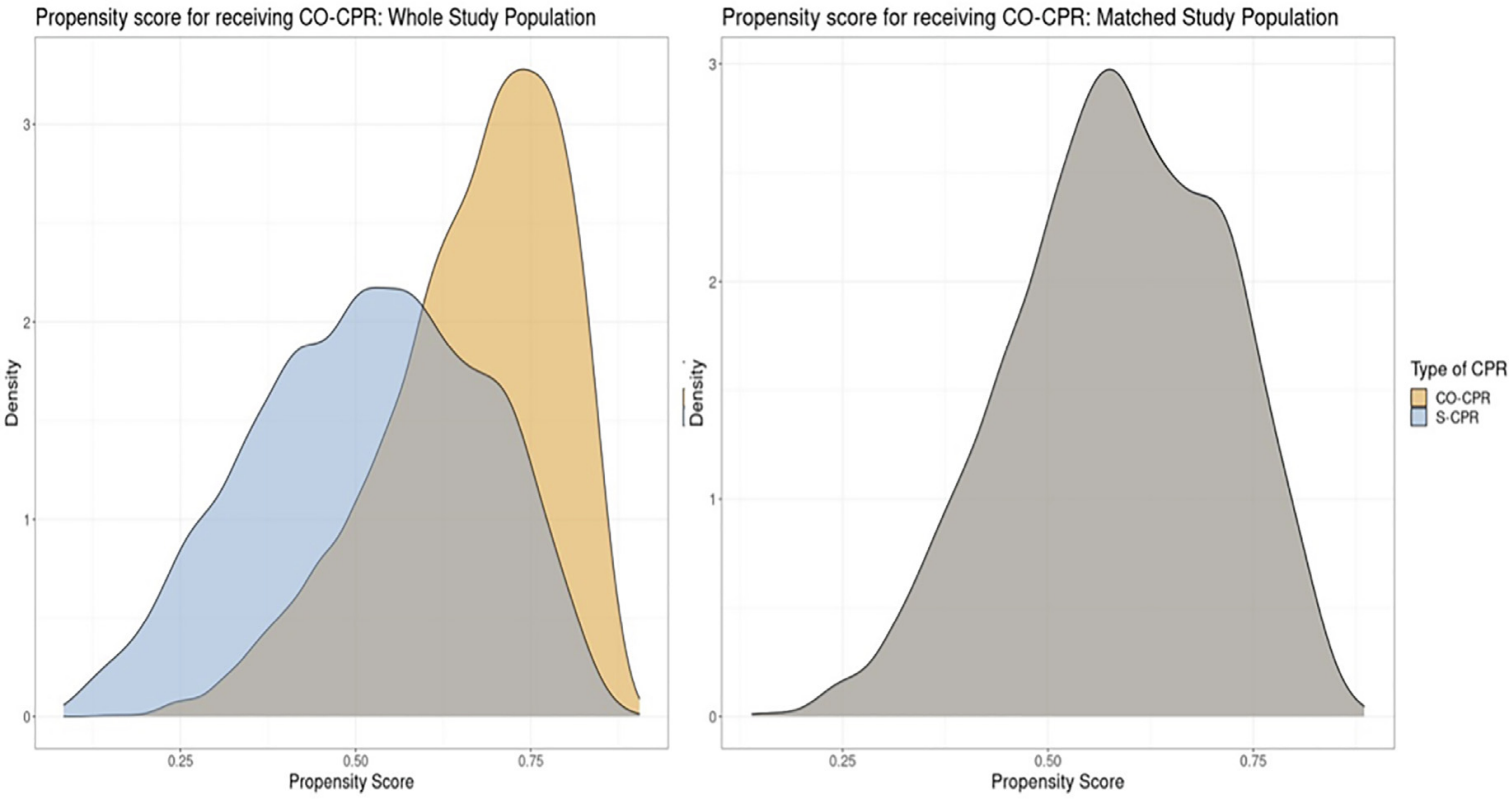
Propensity score for the whole population and the matched population. Two patients with the same propensity score but different treatments are considered ideal for comparing treatment effects. The left panel shows propensity scores in the overall population, which is used to estimate the ATE (i.e., the effect of the treatment in the entire population, and thus the effect that can be expected if all patients were ‘moved’ from untreated to treated. The right panel shows the distribution of propensity scores in the matched cohort, which therefore includes one treated patient who is perfectly matched to one untreated patient; this analysis therefore estimates the treatment effect on those who were actually treated (ATT).

The distribution of propensity scores in the S-CPR and CO-CPR group is presented in Fig. 2. As evident in the figure, the two groups have largely overlapping propensity scores (i.e., there are comparable individuals with differing exposures) and after 1-to-1 matching the propensity scores are perfectly aligned (Fig. 2).

**Fig. 3:**
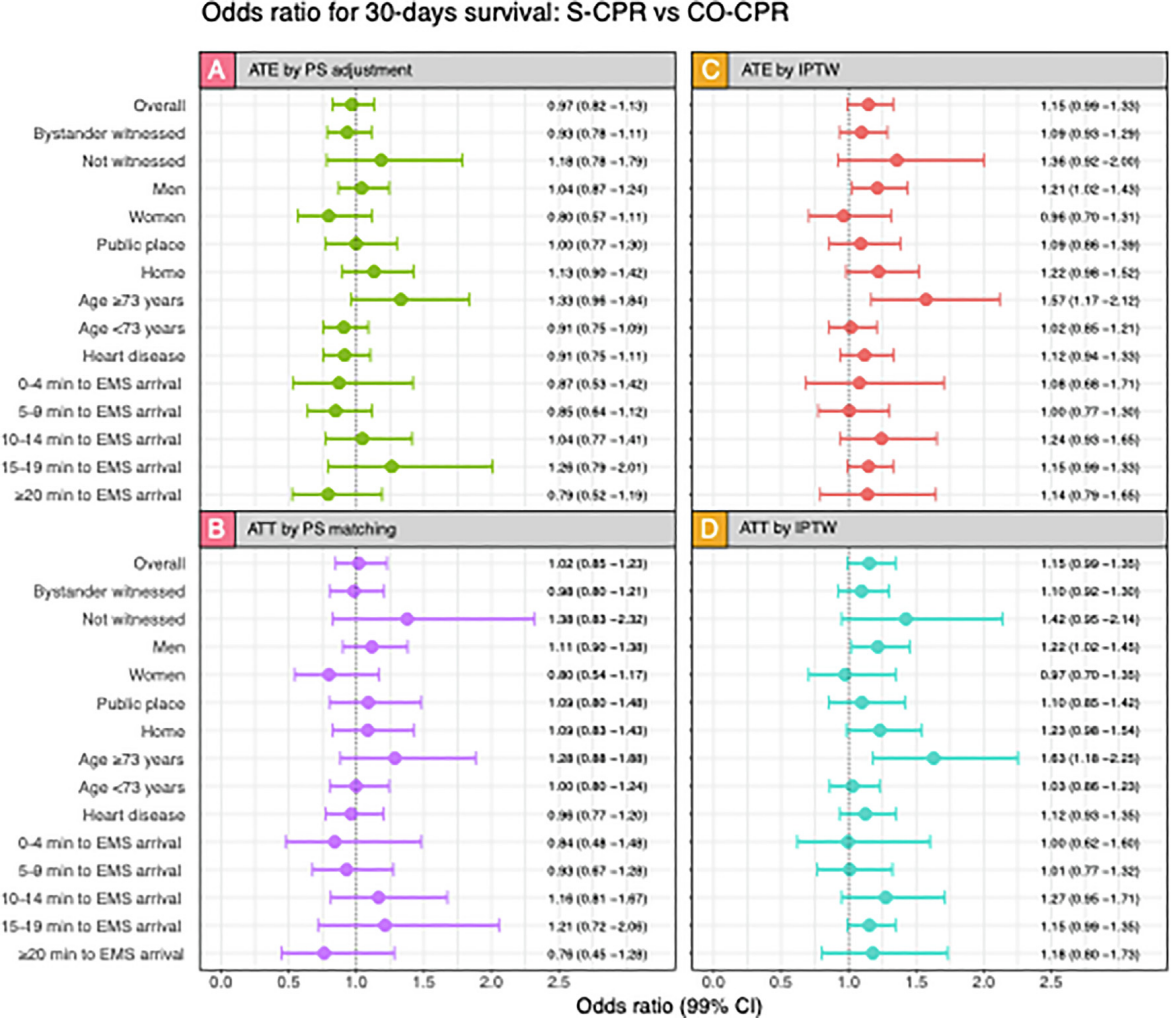
Odds ratio for survival, S-CPR vs CO-CPR in various subgroups at 30 days. A. Shows odds ratio in the whole population (ATE) when adjusting for propensity score. B. Shows odds ratio for the 1–1 matched population (ATT) adjusted for unbalanced variables. C. shows odds ratio in the whole population in the balanced calculation. D. Shows odds ratio in the matched population when using balanced covariates. More detailed description of the analyses can be seen under methods. 99% confidence intervals are shown between the bars.

Probabilities of survival at 30 days after adjusting for propensity score in the whole population (ATE; Fig. 3A) and in the matched study population adjusting for unbalanced variables (DA-CPR, Defibrillator connected by bystander and bystander educational level) and propensity score (ATT; Fig. 3B) are presented in Fig. 3. The results showed no significant differences in any subgroup.

#### Propensity scores for IPTW

Supplementary Table 2 demonstrates that covariates were sufficiently balanced by the propensity score weights. Regarding ATE (Fig. 3C), the subgroup analysis including only men, as well as the analysis including patients aged 73 years or older, we note odds ratios of 1.21 (99% CI 1.02–1.43) and 1.57 (99% CI 1.17–2.12) for S-CPR vs. CO-CPR, respectively. Regarding ATT (Fig. 3D), the subgroup analysis including patients aged 73 years or older displayed an odds ratio of 1.63 (99% CI 1.18–2.25). Remaining odds ratios were non-significant.

## Discussion

The main finding of this study is that bystander CO-CPR is in virtually all analyses non-inferior to bystander S-CPR, with regards to 30-day survival. We studied this association using both an ATE and ATT approach, using propensity scores to perform covariate adjustment, matching and weighting; we did not observe any clear pattern suggesting that S-CPR and CO-CPR differ in terms of survival. The only statistically significant differences were noted for the subgroup of patients aged above the median age (73 years) and in one of four analyses, we noted that men who received S-CPR had slightly higher survival. It is possible that this is a chance finding, despite the use of 99% confidence intervals. Indeed, a confidence interval of 99% corresponds to a p value of 0.01, but a Bonferroni correction would require us to use p values of 0.0008, which would invalidate all significances observed. Hence, we believe our study fails to safely reject the null hypothesis, such that we observe no convincing difference in survival with regards to type of bystander CPR in OHCA.

### Methodological considerations

It is currently believed that the propensity score is the preferred approach for causal inference in observational studies.^15^ As compared with direct covariate adjustment, propensity scores yield, estimates closer to the true values. The vast majority of all propensity score studies use conventional logistic regression to calculate the propensity score15., 16., 17. and then evaluate the performance of the propensity score by checking balance statistics (e.g., baseline differences in a matched subgroup). However, with the recent advances in machine learning we decided to not define a single logistic regression model, which would be prone to our biases and expectations. Instead, we employed a rather comprehensive algorithm that sifted through 12,008 prediction models including the leading frameworks for prediction on structured data. We chose to compute the propensity score both using the model with the highest prediction accuracy, as well as the model yielding the greatest balance of baseline covariates.

We realize that under ideal conditions, we would have used the same model to calculate propensity scores and to estimate relative variable importance. However, this was not justifiable given that we know that conventional XGBOOST, GBM, RF etc., do not provide unbiased estimates of relative importance. The reason for this dilemma is simply that there is currently only one validated software implementation of conditional variable importance, and that is the one available for random forest by Strobl et al.^14^

It is also important to stress the fact that this study is observational in nature. No observational study is unequivocally capable of determining causality. It may be that some sentences in this report may allude to causal effects, but we believe that although propensity score methods have been demonstrated to be highly capable of yielding estimates close to the truth (as defined by randomized trials and simulation studies), they always carry the risk of residual confounding. However, this risk is lower for propensity score approaches, as compared with conventional regression models.^16^

The fact that bystander CPR was not among the most important predictors was an unexpected finding. This is presumably not explained by correlations with other predictors, but it may be explained by *mediation*. It is important to separate mediation from correlation. The effect of one factor may be mediated by another factor, which from a mathematical point of view may appear as the strong predictor. Consider the situation with bystander CPR; receiving bystander CPR would increase the probability of regaining circulation and breathing, such that the effect of bystander CPR may be mediated through those predictors instead. We plan to resolve this by performing a formal mediation analysis in the future.

Unadjusted analyses in our Kaplan Meier plots showed small difference regarding survival in the overall group and among men, indicating that patients receiving S-CPR survive more often (which is presumably driven by the association observed among men). However, the fact that this difference disappeared in the propensity score model, indicates that the difference seen in the Kaplan Meier plot is rather due to one or multiple covariates. Possible covariates are differences in the number of patients suffering CA at home, first recorded rhythm, defibrillation by bystanders, education of bystanders or that the mean age was higher in the CO-CPR group.

### Results in relation to previous studies

Our findings are largely, although not completely, in line with a previous study from SRCR^7^ that showed improved survival in the S-CPR group after adjustment for prognostic factors. This can be due to the fact that we used a different study population and a different modelling approach. However, our main results are consistent with other studies showing no differences regarding survival comparing S-CPR against CO-CPR.18., 19.

There was a tendency (as judged by the point estimates) for improved survival with S-CPR in non-witnessed CA. This could mean that ventilation is more important when the time from CA to start of CPR is longer. The same tendency could be seen when time from CA to EMS arrival became longer. This has been discussed before and are in line with results in previous studies.^7^ It has been suggested that S-CPR may be preferred when the time between CA and CPR is prolonged or when the cause of the arrest is due to hypoxia.^6^

### Strengths and limitations

We used all registry data from all regions of Sweden, meaning that it is representing the entire Swedish population. We decided to include non-witnessed CA, since regardless of witness status, all our patients were treated by a bystander. To illustrate the distribution of witness status we included this variable in Table 1, where no difference could be seen among the two groups. Even though the propensity score was calculated using all appropriate variables available, there is still a risk of residual confounding, meaning that hypothetically there is a chance that there may be variables that we did not have access to, that might yield other results.

## Conclusions

We found no significant difference between the use of S-CPR and CO-CPR before arrival of EMS after OHCA in terms of 30-day survival; CO-CPR was non-inferior in both the ATE and ATT analyses, supporting current recommendations regarding CO-CPR. It is possible that CO-CPR should be recommended on a broader scale, an issue that must be resolved in randomized clinical trials.

## Conflicts of Interest

None of the authors declare any conflicts of interest relevant to this study.

## CRediT authorship contribution statement

**Matilda Jerkeman:** Formal analysis, Writing – original draft. **Peter Lundgren:** Conceptualization, Writing – review & editing. **Elmir Omerovic:** Conceptualization, Writing – review & editing. **Anneli Strömsöe:** Conceptualization, Writing – review & editing. **Gabriel Riva:** Conceptualization, Writing – review & editing. **Jacob Hollenberg:** Conceptualization, Writing – review & editing. **Per Nivedahl:** Conceptualization, Writing – review & editing. **Johan Herlitz:** Conceptualization, Writing – review & editing. **Araz Rawshani:** Supervision, Formal analysis, Writing – original draft, Conceptualization, Writing – review & editing, Methodology.
