# Supplementary material for: Association between type of bystander cardiopulmonary resuscitation and survival in out-of-hospital cardiac arrest: A machine learning study
Source: Resusc Plus. 2022 Jun 14;10:100245. doi: 10.1016/j.resplu.2022.100245 (PMC9207566; doi:10.1016/j.resplu.2022.100245)
Supplement: Supplementary data 1 [file mmc1.docx]

# Supplementary Material

**Supplementary Figure 1**: Survival distribution in all patients, only men and only women. Risk table showing percentage of survivors at certain days after CA. P= log rank p-value. A p-value < 0,01 is considered significant.

**Supplementary Figure 2**: Relative variable importance for survival in OHCA in percent.

**
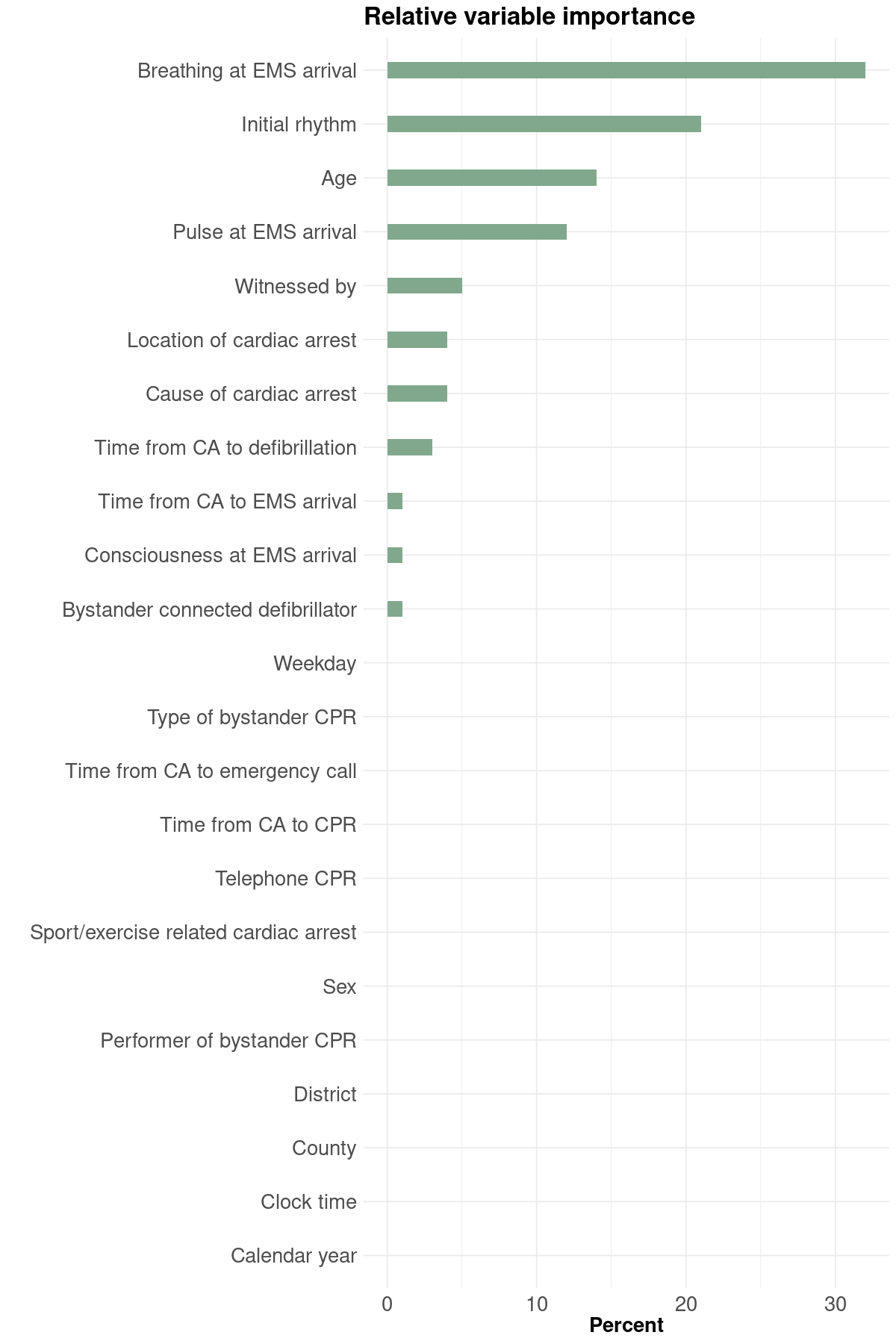
**

**Supplementary Figure 3:** Model performance for the best model in each framework. The best mean accuracy was found in the GBM.

**Supplementary Figure 4**: Graphical summary of GBM models accuracies across the hyperparameter grid.

##

Please note that all continuous variables are scaled and normalized in the table below. This does not affect the interpretation of SMDs.

| ^Supplementary Table 1 – Characteristics of the 1-to-1 matched population^ | | | |
| --- | --- | --- | --- |
|  | ^1^CO-CPR | ^1^S-CPR | ^2^SMD |
| n | 2994 | 2994 |  |
| Age - mean (SD) | -0.01 (1.01) | 0.02 (0.98) | 0.032 |
| Women - n (%) | 983 (32.8) | 1042 (34.8) | 0.042 |
| Calendar year – mean (SD) | -0.05 (0.98) | 0.01 (1.00) | 0.056 |
| Critical time intervals - median (IQR) |  |  |  |
| Time from CA to alarm | -0.12 [-0.15, -0.01] | -0.12 [-0.15, 0.02] | 0.006 |
| Time from CA to CPR | -0.28 [-0.29, -0.21] | -0.26 [-0.29, -0.19] | 0.078 |
| Time from CA to EMS arrival | -0.11 [-0.16, -0.04] | -0.11 [-0.16, -0.05] | 0.031 |
| Time from emergency call to EMS dispatch – median (IQR) | -0.03 (0.78) | -0.01 (0.95) | 0.026 |
| Time from EMS dispatch to EMS arrival | -0.04 (0.47) | 0.00 (1.44) | 0.036 |
| Sports related arrests – n (%) |  |  | 0.051 |
| No | 2602 (86.9) | 2641 (88.2) |  |
| Regular exercise | 365 (12.2) | 336 (11.2) |  |
| Elite sports | 1 (0.0) | 1 (0.0) |  |
| Unknown | 26 (0.9) | 16 (0.5) |  |
| Etiology of cardiac arrest - n (%) |  |  | 0.053 |
| Cardiac disease | 1959 (65.4) | 1953 (65.2) |  |
| Overdose | 102 (3.4) | 94 (3.1) |  |
| Accident/trauma | 48 (1.6) | 42 (1.4) |  |
| Pulmonary disease | 131 (4.4) | 155 (5.2) |  |
| Suffocation | 97 (3.2) | 85 (2.8) |  |
| Suicide | 61 (2.0) | 70 (2.3) |  |
| Drowning | 14 (0.5) | 13 (0.4) |  |
| Other | 582 (19.4) | 582 (19.4) |  |
| Location of cardiac arrest – n (%) |  |  | 0.091 |
| Home | 2105 (70.3) | 2226 (74.3) |  |
| Public place | 606 (20.2) | 521 (17.4) |  |
| Other place | 283 (9.5) | 247 (8.2) |  |
| Witnessed status – n (%) |  |  | 0.064 |
| Non witnessed | 1086 (36.3) | 1127 (37.6) |  |
| Bystander | 1877 (62.7) | 1842 (61.5) |  |
| Ambulance | 11 (0.4) | 16 (0.5) |  |
| Other or combinations | 20 (0.7) | 9 (0.3) |  |
| Bystander CPR – n (%) |  |  | 0.091 |
| No bystander CPR | 35 (1.2) | 36 (1.2) |  |
| CPR by laymen | 2013 (67.2) | 2136 (71.3) |  |
| CPR by professional | 946 (31.6) | 822 (27.5) |  |
| Education of bystander – n (%) |  |  | 0.152 |
| Laymen, no CPR education | 850 (28.4) | 1060 (35.4) |  |
| Laymen, with CPR education | 985 (32.9) | 870 (29.1) |  |
| Professional | 1159 (38.7) | 1064 (35.5) |  |
| ^3^DA-CPR – n (%) | 1696 (56.6) | 1885 (63.0) | 0.129 |
| Defibrillator connected by bystander – n (%) | 319 (10.7) | 194 (6.5) | 0.150 |
| Clock time – mean (SD) | 0.02 (0.99) | 0.00 (1.00) | 0.021 |
| Weekday – n (%) |  |  | 0.053 |
| Monday | 434 (14.5) | 449 (15.0) |  |
| Tuesday | 440 (14.7) | 397 (13.3) |  |
| Wednesday | 395 (13.2) | 425 (14.2) |  |
| Thursday | 401 (13.4) | 384 (12.8) |  |
| Friday | 444 (14.8) | 440 (14.7) |  |
| Saturday | 447 (14.9) | 464 (15.5) |  |
| Sunday | 433 (14.5) | 435 (14.5) |  |
| **^1^** CPR = Cardiopulmonary Resuscitation; S-CPR = standard CPR; CO-CPR = Compression-only CPR  **^2^** SMD = Standardized mean difference. SMDs below 10% (0.1) are considered inconsequential.  ^3^DA-CPR = Dispatcher assisted CPR | | | |

| Supplementary Table 2 – Balance of the study groups using IPTW | | | | |
| --- | --- | --- | --- | --- |
|  | ^1^CO-CPR | ^1^S-CPR | ^2^SES | P-value |
| Age - mean (SD) | 69.5 (16.2) | 69.3 (15.9) | 0.015 | 1.000 |
| Sex: Men - % (SD) | 0.7 (0.5) | 0.7 (0.5) | 0.006 | 0.782 |
| Sex: Women - % (SD) | 0.3 (0.5) | 0.3 (0.5) | -0.006 | 0.782 |
| Year – mean (SD) | 2017.1 (1.4) | 2017.1 (1.4) | 0.036 | 1.000 |
| Time from CA to alarm – mean (SD) | 6.9 (42.8) | 5.3 (17) | 0.038 | 1.000 |
| Time from CA to CPR – mean (SD) | 18.7 (61.9) | 18.2 (61.4) | 0.007 | 1.000 |
| Time from CA to EMS arrival – mean (SD) | 23.2 (95.1) | 22.4 (90.7) | 0.008 | 1.000 |
| Time from call to EMS notified – mean (SD) | 6.2 (73.1) | 5.1 (66.1) | 0.015 | 1.000 |
| Time from call to EMS arrival – mean (SD) | 13.3 (19.6) | 13.7 (24.4) | -0.022 | 1.000 |
| Sports related CA - % (SD) |  |  |  |  |
| No | 0.9 (0.3) | 0.9 (0.3) | -0.030 | 0.218 |
| Regular exercise | 0.1 (0.3) | 0.1 (0.3) | 0.026 | 0.218 |
| Elite sports | 0 (0) | 0 (0) | -0.019 | 0.218 |
| Unknown | 0 (0.1) | 0 (0.1) | 0.022 | 0.218 |
| Cause of CA - % (SD) |  |  |  |  |
| Heart disease | 0.6 (0.5) | 0.7 (0.5) | -0.027 | 0.644 |
| Overdose | 0 (0.2) | 0 (0.2) | 0.031 | 0.644 |
| Accident/Trauma | 0 (0.1) | 0 (0.1) | 0.018 | 0.644 |
| Pulmonary disease | 0 (0.2) | 0 (0.2) | -0.001 | 0.644 |
| Suffocation | 0 (0.2) | 0 (0.2) | 0.002 | 0.644 |
| Suicide | 0 (0.1) | 0 (0.1) | 0.001 | 0.644 |
| Drowning | 0 (0.1) | 0 (0.1) | -0.013 | 0.644 |
| Other | 0.2 (0.4) | 0.2 (0.4) | 0.012 | 0.644 |
| Location of CA - % (SD) |  |  |  |  |
| Home | 0.7 (0.4) | 0.8 (0.4) | -0.036 | 0.117 |
| Public place | 0.2 (0.4) | 0.2 (0.4) | 0.036 | 0.117 |
| Other place | 0.1 (0.3) | 0.1 (0.3) | 0.008 | 0.117 |
| Public location of CA - % (SD) |  |  |  |  |
| Airport | 0 (0) | 0 (0) | 0.001 | 0.445 |
| Amusement park | 0 (0.1) | 0 (0.1) | -0.008 | 0.445 |
| Business center | 0 (0.1) | 0 (0.1) | 0.035 | 0.445 |
| Church | 0 (0) | 0 (0) | 0.023 | 0.445 |
| Home | 0.7 (0.4) | 0.8 (0.4) | -0.036 | 0.445 |
| Other public place | 0 (0.2) | 0 (0.2) | -0.010 | 0.445 |
| Other place | 0.1 (0.3) | 0.1 (0.3) | 0.008 | 0.445 |
| Park/terrain | 0 (0.1) | 0 (0.1) | 0.008 | 0.445 |
| Sports facility | 0 (0.1) | 0 (0.1) | 0.008 | 0.445 |
| Street | 0.1 (0.2) | 0.1 (0.2) | 0.027 | 0.445 |
| Swimming area | 0 (0.1) | 0 (0.1) | 0.000 | 0.445 |
| Train station | 0 (0.1) | 0 (0.1) | 0.016 | 0.445 |
| Water area | 0 (0) | 0 (0) | 0.008 | 0.445 |
| Working place | 0 (0.1) | 0 (0.1) | 0.002 | 0.445 |
| Other location of CA - % (SD) |  |  |  |  |
| Ambulance | 0 (0) | 0 (0) | 0.008 | 0.391 |
| Care facility | 0 (0.2) | 0 (0.2) | 0.014 | 0.391 |
| Dental clinic | 0 (0) | 0 (0) | -0.002 | 0.391 |
| Home | 0.7 (0.4) | 0.8 (0.4) | -0.036 | 0.391 |
| Hospital without ER | 0 (0) | 0 (0) | -0.010 | 0.391 |
| Hotel room | 0 (0) | 0 (0) | 0.020 | 0.391 |
| Other non-public place | 0 (0.2) | 0 (0.2) | 0.000 | 0.391 |
| Primary care clinic | 0 (0) | 0 (0) | -0.015 | 0.391 |
| Private office | 0 (0) | 0 (0) | -0.006 | 0.391 |
| Public place | 0.2 (0.4) | 0.2 (0.4) | 0.036 | 0.391 |
| County - % (SD) |  |  |  |  |
| Stockholm | 0.1 (0.3) | 0.1 (0.3) | 0.016 | 0.997 |
| Uppsala | 0 (0.2) | 0 (0.2) | -0.010 | 0.997 |
| Södermanland | 0 (0.2) | 0 (0.2) | -0.005 | 0.997 |
| Östergötland | 0.1 (0.2) | 0.1 (0.2) | -0.016 | 0.997 |
| Jönköping | 0 (0.2) | 0 (0.2) | 0.005 | 0.997 |
| Kronoberg | 0 (0.1) | 0 (0.1) | 0.016 | 0.997 |
| Kalmar | 0 (0.2) | 0 (0.2) | -0.006 | 0.997 |
| Gotland | 0 (0.1) | 0 (0.1) | 0.001 | 0.997 |
| Blekinge | 0 (0.1) | 0 (0.1) | 0.025 | 0.997 |
| Skåne | 0.1 (0.3) | 0.1 (0.3) | 0.001 | 0.997 |
| Halland | 0 (0.2) | 0 (0.2) | -0.012 | 0.997 |
| Västra Götaland | 0.2 (0.4) | 0.2 (0.4) | -0.013 | 0.997 |
| Värmland | 0 (0.2) | 0 (0.2) | -0.001 | 0.997 |
| Örebro | 0 (0.2) | 0 (0.1) | 0.018 | 0.997 |
| Västmanland | 0 (0.2) | 0 (0.2) | 0.003 | 0.997 |
| Dalarna | 0 (0.2) | 0 (0.2) | -0.005 | 0.997 |
| Gävleborg | 0 (0.2) | 0 (0.2) | -0.001 | 0.997 |
| Västernorrland | 0 (0.2) | 0 (0.2) | -0.007 | 0.997 |
| Jämtland | 0 (0.1) | 0 (0.1) | 0.018 | 0.997 |
| Västerbotten | 0 (0.2) | 0 (0.2) | 0.006 | 0.997 |
| Norrbotten | 0 (0.2) | 0 (0.2) | -0.007 | 0.997 |
| District - % (SD) |  |  |  |  |
| 0 | 0 (0) | 0 (0) | 0.025 | 1.000 |
| 1 | 0 (0.1) | 0 (0.1) | 0.007 | 1.000 |
| 5 | 0 (0.2) | 0 (0.2) | -0.005 | 1.000 |
| 6 | 0 (0.2) | 0 (0.2) | 0.000 | 1.000 |
| 7 | 0 (0.2) | 0 (0.2) | 0.001 | 1.000 |
| 8 | 0 (0.2) | 0 (0.2) | 0.001 | 1.000 |
| 9 | 0 (0.1) | 0 (0.1) | -0.001 | 1.000 |
| 11 | 0 (0.2) | 0 (0.2) | -0.011 | 1.000 |
| 12 | 0.1 (0.3) | 0.1 (0.3) | -0.011 | 1.000 |
| 13 | 0 (0.2) | 0 (0.2) | -0.005 | 1.000 |
| 15 | 0 (0.2) | 0 (0.2) | -0.012 | 1.000 |
| 16 | 0 (0.2) | 0 (0.2) | -0.005 | 1.000 |
| 17 | 0 (0.1) | 0 (0.1) | 0.008 | 1.000 |
| 18 | 0 (0.1) | 0 (0.1) | 0.001 | 1.000 |
| 19 | 0 (0.2) | 0 (0.2) | -0.005 | 1.000 |
| 20 | 0 (0.1) | 0 (0.1) | 0.027 | 1.000 |
| 21 | 0 (0.1) | 0 (0.1) | 0.017 | 1.000 |
| 22 | 0 (0.1) | 0 (0.1) | -0.003 | 1.000 |
| 23 | 0 (0.1) | 0 (0.1) | -0.010 | 1.000 |
| 24 | 0 (0.1) | 0 (0.1) | -0.004 | 1.000 |
| 25 | 0 (0.1) | 0 (0.1) | 0.007 | 1.000 |
| 26 | 0 (0.1) | 0 (0.1) | 0.014 | 1.000 |
| 27 | 0 (0.1) | 0 (0.1) | -0.002 | 1.000 |
| 28 | 0 (0.2) | 0 (0.2) | -0.001 | 1.000 |
| 29 | 0 (0.2) | 0 (0.2) | -0.007 | 1.000 |
| 30 | 0 (0.1) | 0 (0.1) | -0.002 | 1.000 |
| 31 | 0 (0.2) | 0 (0.2) | -0.006 | 1.000 |
| 32 | 0 (0.1) | 0 (0.1) | -0.001 | 1.000 |
| 33 | 0 (0.1) | 0 (0.1) | 0.014 | 1.000 |
| 34 | 0 (0.1) | 0 (0.1) | 0.001 | 1.000 |
| 35 | 0 (0.2) | 0 (0.2) | -0.001 | 1.000 |
| 36 | 0 (0.2) | 0 (0.2) | -0.010 | 1.000 |
| 37 | 0.1 (0.2) | 0.1 (0.2) | -0.016 | 1.000 |
| 38 | 0 (0.1) | 0 (0.1) | 0.018 | 1.000 |
| 39 | 0.1 (0.2) | 0 (0.2) | 0.013 | 1.000 |
| 40 | 0 (0.2) | 0 (0.2) | 0.012 | 1.000 |
| 41 | 0.1 (0.2) | 0.1 (0.2) | 0.002 | 1.000 |
| 43 | 0 (0.1) | 0 (0.1) | 0.017 | 1.000 |
| 44 | 0 (0.1) | 0 (0.1) | 0.018 | 1.000 |
| 45 | 0 (0.2) | 0 (0.2) | 0.003 | 1.000 |
| Witnessed by - % (SD) |  |  |  |  |
| No one | 0.4 (0.5) | 0.4 (0.5) | 0.029 | 0.428 |
| Bystander | 0.6 (0.5) | 0.6 (0.5) | -0.027 | 0.428 |
| Ambulance | 0 (0.1) | 0 (0.1) | -0.008 | 0.428 |
| Other or combinations | 0 (0.1) | 0 (0.1) | -0.005 | 0.428 |
| Bystander CPR - % (SD) |  |  |  |  |
| No bystander CPR | 0 (0.1) | 0 (0.1) | 0.018 | 0.571 |
| CPR by laymen | 0.7 (0.4) | 0.7 (0.4) | -0.012 | 0.571 |
| CPR by professional | 0.3 (0.4) | 0.3 (0.4) | 0.007 | 0.571 |
| Bystander educational level - % (SD) |  |  |  |  |
| Laymen, no CPR education | 0.4 (0.5) | 0.4 (0.5) | 0.030 | 0.266 |
| Laymen, CPR educated | 0.2 (0.4) | 0.3 (0.4) | -0.026 | 0.266 |
| Professional | 0.3 (0.5) | 0.3 (0.5) | -0.008 | 0.266 |
| ^3^DA-CPR: No - % (SD) | 0.3 (0.5) | 0.3 (0.5) | -0.017 | 0.388 |
| ^3^DA-CPR: Yes - % (SD) | 0.7 (0.5) | 0.7 (0.5) | 0.017 | 0.388 |
| Defibrillator connected by bystander: No - % (SD) | 0.9 (0.3) | 0.9 (0.3) | 0.009 | 0.589 |
| Defibrillator connected by bystander: Yes - % ((SD) | 0.1 (0.3) | 0.1 (0.3) | -0.009 | 0.589 |
| Clock Time – mean (SD) | 11.2 (6.9) | 11.3 (6.8) | -0.015 | 1.000 |
| Weekday - % (SD) |  |  |  |  |
| Monday | 0.1 (0.4) | 0.2 (0.4) | -0.010 | 0.991 |
| Tuesday | 0.1 (0.4) | 0.1 (0.3) | 0.013 | 0.991 |
| Wednesday | 0.1 (0.3) | 0.1 (0.3) | -0.007 | 0.991 |
| Thursday | 0.1 (0.3) | 0.1 (0.3) | 0.009 | 0.991 |
| Friday | 0.1 (0.4) | 0.1 (0.4) | 0.001 | 0.991 |
| Saturday | 0.1 (0.4) | 0.1 (0.4) | -0.004 | 0.991 |
| Sunday | 0.1 (0.4) | 0.1 (0.4) | -0.003 | 0.991 |
| **^1^** CPR = Cardiopulmonary Resuscitation; S-CPR = standard CPR; CO-CPR = Compression-only CPR  **^2^** SES = Standardized Effect size. SESs below 10% (0.1) are considered inconsequential.  ^3^DA-CPR = Dispatcher assisted CPR. | | | | |

# Statistical approaches

**Approach 1: Calculation of propensity scores using the model with highest accuracy for type of bystander CPR**

This approach included the following four steps: (1) data pre-processing; (2) selection of frameworks, model building and hyperparameter tuning; (3) estimation of propensity score using the best model – the propensity score was used as a covariate and, additionally, as a matching instrument to perform 1-to-1 matching; (4) calculation of odds ratios for 30-days survival in relation to type of bystander CPR.

**Data pre-processing** – Variables with zero or near-zero variance display no or very little variation, respectively, among the observations. Such variables may introduce noise and reduce the accuracy of a prediction model. We searched for zero variance and near-zero variance variables to exclude them, however, no such variables were identified. In order to exclude redundant variables, we assessed the correlations among all predictors (categorical variables were converted into numerical before calculation of correlations). We intended to exclude variables with correlations exceeding 0.75, although no such variables were present in the dataset (the maximum correlation observed was 0.53). All continuous variables were scaled (*i.e.,* the value was divided by the overall standard deviation) and normalized (*i.e.,* the overall mean was subtracted from the value). Finally, we used *k-nearest neighbor (KNN) imputation* to complete the imputation of a few values that were still missing after the MICE procedure.
 **Model building** – We used a semi-automated approach to compute and evaluate a large number of prediction models, all of which predict type of bystander CPR. Models were built using the following frameworks: Gradient Boosting Machine (GBM), Support Vector Machine (SVM), Random Forest (RF), Logistic Regression (GLM [Generalized Linear Model]), Extreme Gradient Boosting (XGBOOST) and neural networks (Deep Learning). For SVM and GLM we computed one model each. For GBM we constructed a hyperparameter grid with the following specifications: max tree depth: 3, 4, 6, 8, 10, 12, 14; number of trees: 100, 200, 300, 400, 500, 600, 700, 800, 1000; shrinkage (learning rate): 0.001, 0.01; minimal terminal node size: 4, 8, 10, 12, 16. For XGBOOST we constructed a hyperparameter grid with the following specifications: max tree depth: 3, 4, 5, 6, 8, 10; number of trees: from 50 to 700, by steps of 50; shrinkage; 0.025, 0.05, 0.1, 0.3, 0.5; minimum loss reduction: 0.5, 0.75, 1.5; subsample ratio of columns: 0.6, 0.8, 1.0; minimum sum of instance weight: 0.5, 1.0, 1.5; subsample percentage: 0.6, 0.8, 1.0. For RF we used 1000 trees and tried 3, 4, 6, 8, 10 and 12 variables in each model. Neural networks were constructed with 1 to 10 hidden layers, with weight decay between 0, 0.05, 0.1, 1 and 2. In total we built 12,008 prediction models. All models were evaluated using 5-fold cross validation, repeated 5 times. The best model was defined as the model with the highest average accuracy and was used to calculate propensity score for type of bystander CPR. The variables included to estimate the propensity score were as follows: age, sex, calendar year, time from CA to alarm, time from CA to CPR start, time from CA to EMS arrival, time from alarm to alert, time from alert to EMS arrival, sports related CA, reason for CA, location, location in a public place, location in other place, county, district, type of witness, bystander’s profession, bystander’s educational level, DA-CPR, defibrillator connected by bystander, clock time and weekday.
 **Estimation of propensity score** – We used the best model, among 12,008 models evaluated, to calculate propensity scores for each individual. A propensity score is a score measuring each individual´s probability of being exposed, which, in this study is exposure to CO-CPR, given the person’s baseline characteristics. Two individuals with the same propensity scores have the same probability of being exposed (*i.e.,* treated with CO-CPR); if they receive different treatments, then they provide an opportunity to compare the treatments. Indeed, two people with identical propensity scores can be matched and thus simulate a randomized clinical trial (11).
 **The association between type of bystander CPR and 30-days survival** – We used logistic regression to study how type of bystander CPR related to 30-days survival. The regression model was computed in the overall cohort, which estimates the average treatment effect (ATE) and in a 1-to-1 matched cohort, which estimates the average treatment effect on those actually treated. In the overall cohort we used the propensity score as a covariate and expanded it into a restricted cubic spline with 5 knots, which allows for relaxing of linearity assumptions. In the matched study population (ATT analysis) we only included baseline covariates which were not adequately balanced by the matching procedure.

**Approach 2: Using propensity score to calculate the inverse probability of treatment weighting (IPTW)**

The IPTW approach aims to balance the distribution of baseline covariates in the CO-CPR and S-CPR group by using weights based on the propensity score. Each observations weight is equal to the inverse probability of receiving the treatment actually received (12)***.*** The weights are then used in the logistic regression model to estimate odds ratios for ATE and ATT (13)***.*** We used GBM, with automatic grid search across 2000 models, to identify the model yielding the greatest balance in baseline covariates (14). The purpose of this approach was to check the robustness of our inferences across several methods. In order to account for multiple testing, we used 99% confidence intervals for all odds ratios.
